# Protective Effect of the Polysaccharides from *Taraxacum mongolicum* Leaf by Modulating the p53 Signaling Pathway in H22 Tumor-Bearing Mice

**DOI:** 10.3390/foods11213340

**Published:** 2022-10-24

**Authors:** Pei Chen, Yi Chen, Zhi-Qian Yan, Su-Yun Ding, Hui-Ping Liu, Jian-Qiu Tu, Xiao-Wei Zhang

**Affiliations:** State Key Laboratory of Food Nutrition and Safety, Ministry of Education of China, College of Food Engineering and Biotechnology, Tianjin University of Science &Technology, Tianjin 300457, China

**Keywords:** dandelion leaf polysaccharides, antitumor activity, *in vivo*

## Abstract

Dandelion is an edible plant with a variety of bioactive components. This paper mainly reports the antitumor activity of dandelion polysaccharide DLP120 on H22 tumor-bearing mice. DLP120 is an acidic polysaccharide composed of pectin and arabinogalactan. The results indicate that DLP120 markedly inhibited tumor growth in a dose-dependent manner and attenuated and regulated negative effects on organs. In addition, DLP120 not only increased the viability of spleen lymphocytes and natural killer (NK) cells, but also increased the proportion of lymphocyte subsets in peripheral blood. Furthermore, Hematoxylin-Eosin (HE) staining showed that tumor tissues and cells exhibited typical pathology features. Annexin V FITC/PI staining and cell cycle distribution results further confirmed apoptosis and cell cycle arrest in S and G2 phases. Notably, there was a significant accumulation of reactive oxygen species. Western blotting results demonstrated that the expression of p53 was up-regulated in the DLP120 group. Moreover, the pro-apoptotic protein Bax was up-regulated while the inhibitory-apoptotic protein Bcl-2 was down-regulated. In addition, the expression of Fas and FasL, associated with the death receptor pathway, were also up-regulated. Overall, administration of DLP120 in H22 tumor-bearing mice can not only enhance immunity but also directly induce tumor cell apoptosis.

## 1. Introduction

Hepatocellular carcinoma (HCC) has received much attention due to its high morbidity and mortality, especially in Asia and Africa [1,2]. At present, the radical treatment methods for HCC are mainly surgical resection and liver transplantation, but most patients do not meet the treatment conditions [3,4]. For chemotherapy patients, they not only have to suffer pain but also the prognosis is not ideal [5]. Natural products have complex biological activities, and they can work synergistically to exert their effects while reducing adverse reactions [6]. Recently, active ingredients extracted from natural products have received extensive attention due to their low toxicity and high biological activity. For example, *G. frondosa* polysaccharides can not only enhance immunity against tumor cell development, but also directly induce tumor cell apoptosis. In addition, it can act as a biological response modifier to help the host withstand adverse biological stress [7].

Dandelion (*Taraxacum mongolicum Hand.-Mazz.*) is a perennial herb of the Asteraceae family [8]. Dandelion leaves are a dietary source with a variety of bioactive components (sesquiterpenoid, triterpenoid, phytosterol, flavonoids and phenolic acids, etc.) that have health-promoting benefits [8,9]. Dandelion is widely used in food and medicinal applications. As a edible vegetable, the diuretic effect of dandelion leaf is consistently stronger than that of the root [8]. Studies have revealed that dandelion leaf supplementation can increase the activity of antioxidant-related enzymes in the liver of rabbits fed with a high-cholesterol diet [10]. In addition, dandelion leaf extract exerts an anti-inflammatory effect by inhibiting the expression of iNOS and COX-2 [11]. Currently, dandelion polysaccharides have become the object of interest for a variety of biologically active activities. Multiple studies have reported the immune and antitumor activities of dandelion polysaccharides [12,13,14]. Although the health benefits of dandelion polysaccharides have been well established, there are still underutilized polysaccharide components worthy of study. The biological activity of polysaccharides is associated with their structural characteristics, which depends upon extraction conditions. The extraction of dandelion polysaccharides is mostly by hot water extraction, but no higher than at 100 °C. Therefore, this study mainly revolves around dandelion leaf polysaccharides (DLP120) extracted at 120 °C to obtain the fraction composed of pectin and arabinogalactan. There are few reports on pectin-arabinogalactan polysaccharides from dandelion. Surprisingly, we found that DLP120 has the potential to inhibit the growth of human hepatoma HepG2 cells in vitro. This study aimed to elucidate the antitumor effect of DLP120 on H22 tumor-bearing mice *in vivo*, and provide a new direction for anti-HCC drug adjuvants.

## 2. Materials and Methods

### 2.1. Materials

Dandelion leaves were obtained from northeastern dandelions in the Greater Xing’an Mountains. H22 hepatoma cells were provided by Solomon Biotechnology Co., Ltd. (Tianjin, China). The 5-Fu, Annexin V-FITC/PI apoptosis detection kit, cell cycle detection kit, Reactive Oxygen Species assay kit, Concanavalin A (ConA), lipopolysaccharide (LPS), Hematoxylin-Eosin staining kit and 3- (4, 5-dimethylthiazol-2-yl) -2, 5-diphenyltetrazolium bromide (MTT) were bought from Solarbio Biotechnology Co., Ltd. (Beijing, China). Antibodies against p53, Fas, FasL, Bax, Bcl-2, β-Actin, FITC anti-mice CD3+, PE anti-mice CD4+, FITC anti-mice CD8+ and PE anti-mice CD19+ were purchased from Biolegend Biotech Co., Ltd. (Shanghai, China). All of the other chemical reagents were analytical grade.

### 2.2. Preparation of DLP120

**Figure 1:**
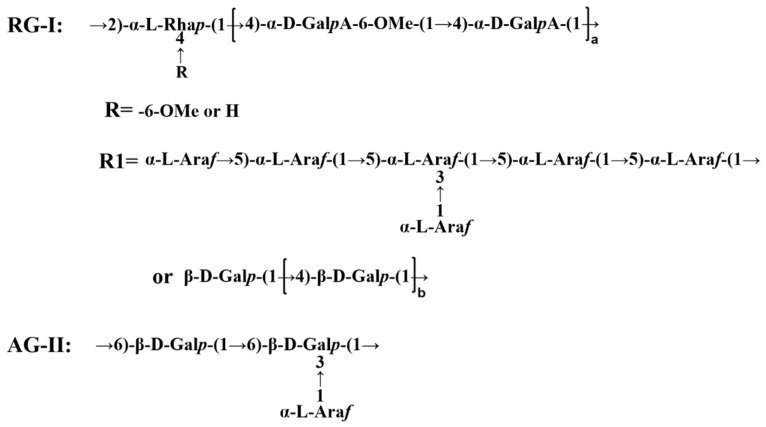
Structural model of DLP120. Note: RG-Ⅰ stands for rhamnogalacturonan Ⅰ. AG-Ⅱ stands for arabinogalactan Ⅱ.

The preparation of dandelion leaf polysaccharides followed our previous experiments. Briefly, dandelion leaf powder was extracted at 120 °C in hot water for 2 h with a solid-liquid ratio of 1:20. The solution was concentrated to one fifth of its original volume by rotary vacuum. The polysaccharide was precipitated by adding ethanol to a final concentration of 60% overnight at 4 °C. The obtained polysaccharide was deproteinized using the Seveg method [15]. The resulting solution was dialyzed using a 10 kDa dialysis bag and purified with Sephadex G-200 to harvest the polysaccharide fraction DLP120. A structural model of DLP120 is shown in Figure 1.

### 2.3. Design of Animal Model

Sixty female Kunming mice (7–8 weeks old, body weight 20 ± 2 g) were purchased from SiPeiFu Biotechnology Co., Ltd. (Beijing, China) with license number SCXK (jing) 2019-0010. All animal experimental procedures strictly followed the “Regulations on the Administration of Laboratory Animals” (China). These mice were kept at 20–25 °C and 45–55% humidity on a 12-h light/12-h dark cycle with free access to food and water. After one week of acclimatization, the mice were randomly divided into six groups: blank group, model group, 5-Fu group (positive control), 100 mg/kg DLP120 group, 200 mg/kg DLP120 group and 300 mg/kg DLP120 group. The blank group, model group and positive control group were intragastrically administered 0.2 mL of normal saline once a day for 14 days, while the DLP120 group was given an equal volume of gavage. Subsequently, except for the blank group, all mice were injected with H22 hepatoma cells (2 × 10^6^ cells/mL) subcutaneously in the axilla of the right forelimb. The blank group, model group and DLP group were treated as before, while the positive control group was intraperitoneally injected with 5-Fu (20 mg/kg) 0.2 mL once a day for another 14 days.

### 2.4. Immune Organs Indices and Inhibitory Rates

All mice were weighed and sacrificed by cervical dislocation. The spleen, thymus, liver and tumor were removed and weighed. The immune organ index is the ratio of the corresponding immune organ to body weight. The tumor inhibition rate was calculated according to the following formula:

$$\mathrm{Tumor}\mathrm{inhibition}\mathrm{rate}\left(\%\right)=\left(W_{1}-W_{2}\right)/W_{1}\times 100$$

where W_1_ is the mean tumor weight of model group, W_2_ is the mean tumor weight of DLP120 groups and 5-Fu.

### 2.5. NK Cells Activities

NK cells were isolated by an NK cell isolation kit according to the manufacturer’s instructions. The activities of NK cells were detected following a method in the literature [16]. NK cells and H22 cells served as effector and target cells, respectively. Experimental groups were added to 100 μL each of the effector cells and target cells at a ratio of 20:1 in a 96-well plate. Two control groups were added to 100 μL of effector cells and target cells, respectively. Then, DMEM complete medium (100 μL) was added to each control group. After incubating at 37 °C in a 5% CO_2_ completely humidified environment for 48 h, the optical density (OD) of NK cells and H22 cells was determined by the MTT method [17] using a microplate reader (Bio-Rad, Hercules, CA, USA). NK cytotoxic activities were calculated using the following formula:

$$\mathrm{NK}\mathrm{cytotoxic}\mathrm{activity}\left(\%\right)=\left({\mathrm{OD}}_{0}+{\mathrm{OD}}_{1}-{\mathrm{OD}}_{2}\right)/{\mathrm{OD}}_{0}\times 100$$

where OD_0_ is the OD value of target cells, OD_1_ is the OD value of effector cells, and OD_2_ is the OD value of the effector-to-target cells.

### 2.6. Splenic Lymphocyte Proliferation Activity

The dissected spleen was ground to obtain a homogeneous cell suspension. Spleen lymphocyte suspension was prepared following the previous method [18]. The cells were adjusted to 1×10^7^ cells/mL and seeded in a 96-well plate with ConA (5 μg/mL) or LPS (10 μg/mL) each well. An equal volume of DMEM complete medium was added to the control wells. Cells were cultured in a 5% CO_2_ incubator at 37 °C for 48 h. MTT (5 mg/mL) was added and continuously incubated for 4 h in the dark. Absorbance was measured at 570 nm using a microplate reader (Bio-Rad, Hercules, CA, USA). The stimulation index (SI) was calculated using the following formula:

$$\mathrm{Stimulation}\mathrm{index}\left(\mathrm{SI}\right)={\mathrm{OD}}_{1}/{\mathrm{OD}}_{2}$$

where OD_1_ is the OD value of splenic lymphocytes exposed to Con A or LPS, OD_2_ is the OD value of DMEM medium.

### 2.7. Peripheral Lymphocyte Subsets

Blood was collected from the tail vein of mice. Red blood cell lysate was added, followed by centrifugation to harvest cells. This was repeated 2–3 times until lysis was complete. The resulting cell suspension was placed in two test tubes. Monoclonal antibodies (CD8-FITC, CD4-PE, CD3-FITC, CD19-PE) were added into the two tubes and incubated in the dark for 30 min. The distribution of lymphocyte subsets was detected by flow cytometry (Becton Dickinson, Franklin Lakes, NJ, USA).

### 2.8. Hematoxylin-Eosin Staining

#### 2.8.1. Mice Solid Tumors

Tumor tissue was placed it in 4% paraformaldehyde fixative solution for 4 h. After rinsing with running water, it was dehydrated with graded alcohol. Xylene was added to make the tissue transparent. After paraffin embedding, the samples were sectioned and placed on glass slides. Next, tissue sections were stained according to the instructions of the Hematoxylin-Eosin staining kit. The tumors were placed under a microscope (Nikon, Tokyo, Japan) for observation and photography.

#### 2.8.2. Mice Tumor Cells 

Tumor cells were collected by grinding and the grinding fluid obtained. After adjusting the density, the tumor cells were stained with Hematoxylin-Eosin staining kit. The cells were observed by a microscope (Nikon, Tokyo, Japan).

### 2.9. Tumor Cell Cycle Assay

Tumor cell suspensions were prepared by grinding tumor tissue. They were washed twice with ice-cold phosphate buffered saline (PBS), followed by centrifugation to collect cells. Next, pre-chilled 70% ethanol was used to fix the cells for 12 h. Propidium iodide staining solution was prepared according to cell cycle and apoptosis analysis kit instructions and stained for 30 min at 37 °C in the dark for analysis by flow cytometry (Becton Dickinson, Franklin Lakes, NJ, USA).

### 2.10. Annexin V/PI Assay

The collected tumor cell suspension was stained according to the instructions of the Annexin V-FITC/PI apoptosis detection kit. Briefly, cell density was adjusted to 1×10^6^ cells/mL and added (100 μL) to flow tubes. Annexin V-FITC (5 μL) was added for 10 min followed by PI staining (5 μL) for 5 min. The resulting cells were analyzed by flow cytometry (Becton Dickinson, Franklin Lakes, NJ, USA) within 1 h.

### 2.11. Reactive Oxygen Species (ROS) Determination

Tumor cells were treated with a 10 μM 2’, 7’-dichlorofluorescent yellow diacetate (DCFH-DA) probe solution, which was dissolved with PBS, following by 30 min at 37 °C in the dark. Reactive oxygen species were determined using the assay kit by flow cytometry (Becton Dickinson, Franklin Lakes, NJ, USA).

### 2.12. Western Blot (WB) Analysis

RIPA lysis buffer was added to the tumor cells to fully lyse the protein. The supernatant was collected to harvest tumor cell protein samples. Protein concentrations were determined by the BCA method [19]. Protein samples were completely separated by a 12% resolving gel and a 5% stacking gel. The separated target proteins were transferred using a PVDF membrane, following by placing in 5% BSA solution to block for 2 h. Subsequently, β-Actin, p53, Bax, Bcl-2, Fas and FasL were diluted according to the manufacturer’s instructions and incubated with the membrane for 12 h at 4 °C. Furthermore, antibodies were incubated with the corresponding horseradish peroxidase (HRP)-conjugated secondary antibodies for 2 h at room temperature. Proteins were detected using enhanced chemiluminescence (ECL) detection kits to prepare fluorescent colors.

### 2.13. Statistical Analysis

Data are expressed as mean ± standard deviation (SD). Statistical significance of differences was determined using Student’s t-test and one-way ANOVA. Data was considered significant at *p* < 0.05.

## 3. Results and Discussion

### 3.1. Effects of DLP120 on Tumor Growth and Immune Organs

Except for the blank group, tumor tissue was observed in other groups, indicating that the animal experimental model was successfully established. The H22 tumor-bearing mice model was established to evaluate the antitumor activity of DLP120, while 5-Fu was used as a positive control. As shown in Table 1, the 5-Fu group and the DLP120 group could effectively inhibit the growth of mice tumors compared with the model group. The tumor inhibition rate of 5-Fu reached 67.95%, while the tumor inhibition rates of different doses of DLP120 were 18.75, 49.52, and 59.64%, respectively. This indicates that DLP120 could dose-dependently inhibit the proliferation of H22 solid tumors. There was no significant difference in the body weight of mice in the model group and the DLP120 group compared with the blank group. This may be due to the weight of the tumor. Conversely, the body weight of mice in the 5-Fu group was dramatically lower than that in the model group. It is worth noting that two mice died in the 5-Fu group after the administration. This suggested that 5-Fu have side effects while effectively inhibiting growth.

**Figure 2:**
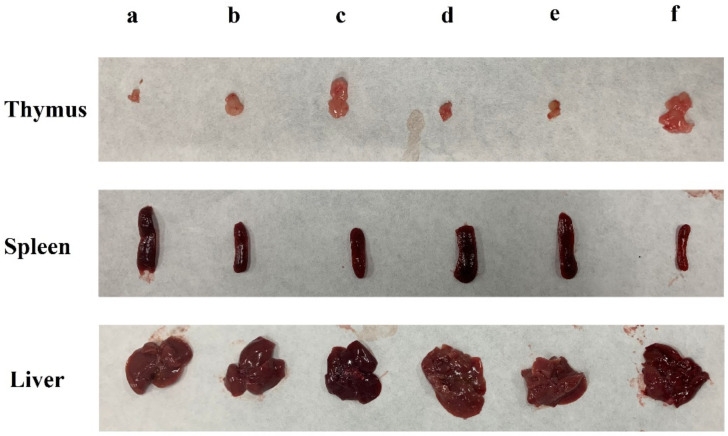
Effects of DLP120 on the immune system organs of H22-bearing mice. (**a**) DLP120 100 mg/kg; (**b**) DLP120 200 mg/kg; (**c**) DLP120 300 mg/kg; (**d**) 5-Fu group; (**e**) model group; (**f**) blank group.

The liver is the main organ for detoxification [20]. Compared with the blank group, the liver indexes of the other groups increased to varying degrees (*p* < 0.05). However, treatment with all doses of DLP120 significantly decreased the liver index (*p* < 0.05) compared with the 5-Fu group. There was no significant difference between the DLP120 group and the model group (Table 1). It can also be seen from Figure 2 that transplant of the H22 solid tumor caused enlargement of the liver and a certain degree of liver damage. The spleen and thymus produce lymphocytes and are critically important to the immune system [21,22]. As shown in Figure 2, compared with the blank group, the spleen was obviously swollen, and the thymus was dramatically degenerated in tumor-bearing mice. All of the tumor groups presented a significant increase of the spleen index and decrease in the thymus index compared with the blank group (*p* < 0.05). The DLP120 group dose-dependently decreased the spleen index while increasing the thymus index. The thymus index of mice was not significantly different from that of the blank group at a dose of 300 mg/kg DLP120. In short, DLP120 can effectively protect the spleen and thymus by alleviating the damage caused by H22 solid tumors to immune system organs, and also inhibits tumor growth.

### 3.2. Effect of DLP120 on Activities of NK Cells

**Figure 3:**
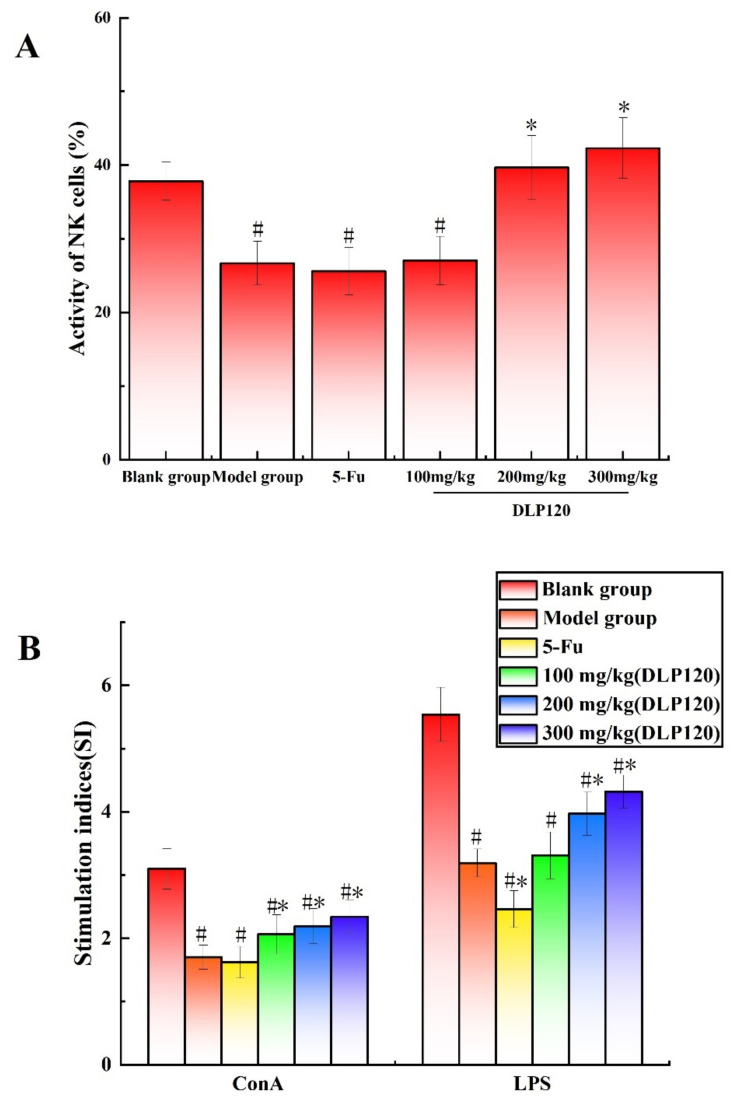
Effects of DLP120 on NK cells (**A**) and lymphocytes (**B**) of H22 tumor-bearing mice. Note: ^#^ *p* < 0.05, compared to blank group; * *p* < 0.05, compared to model group.

Activated NK cells, as immune system cells, can secrete and synthesize a variety of cytokines to directly kill target cells, and they play an immunomodulatory role [23]. Therefore, the activity of NK cells can be used an important indicator of the immune functions. The results showed (Figure 3A) that the killing activity of NK cells in the blank group was distinctly higher than that in the model group and 5-Fu (*p* < 0.05). However, the activity of NK cell was remarkably increased in the 200 and 300 mg/kg DLP120 groups compared to the model group (*p* < 0.05). This suggests that DLP120 can enhance spleen NK cell activity to effectively inhibit tumor growth.

### 3.3. Effect of DLP120 on Splenic Lymphocyte Proliferation

The spleen is an important lymphoid organ, including B lymphocytes and T lymphocytes. The ability of lymphocytes to respond to mitogens or specific antigens reflects the immune potential of the organism [24]. Lymphocytes were stimulated to proliferate using ConA or LPS to confirm the effect of DLP120 on lymphocytes. As the results in Figure 3B shown, DLP120 with ConA and LPS can stimulate splenic lymphocyte proliferation with a dose of 200 mg/kg to 300 mg/kg compared to the model group (*p* < 0.05). There was no significant difference in the proliferation ability of spleen lymphocytes in the 5-Fu group compared with the model group regarding the ConA, while the LPS stimulation index was lower (*p* < 0.05). Thus, DLP120 can effectively stimulate the proliferation of spleen lymphocytes to protect immune function in a certain extent.

### 3.4. Effect of DLP120 Peripheral Lymphocytes Subsets Distributions

**Figure 4:**
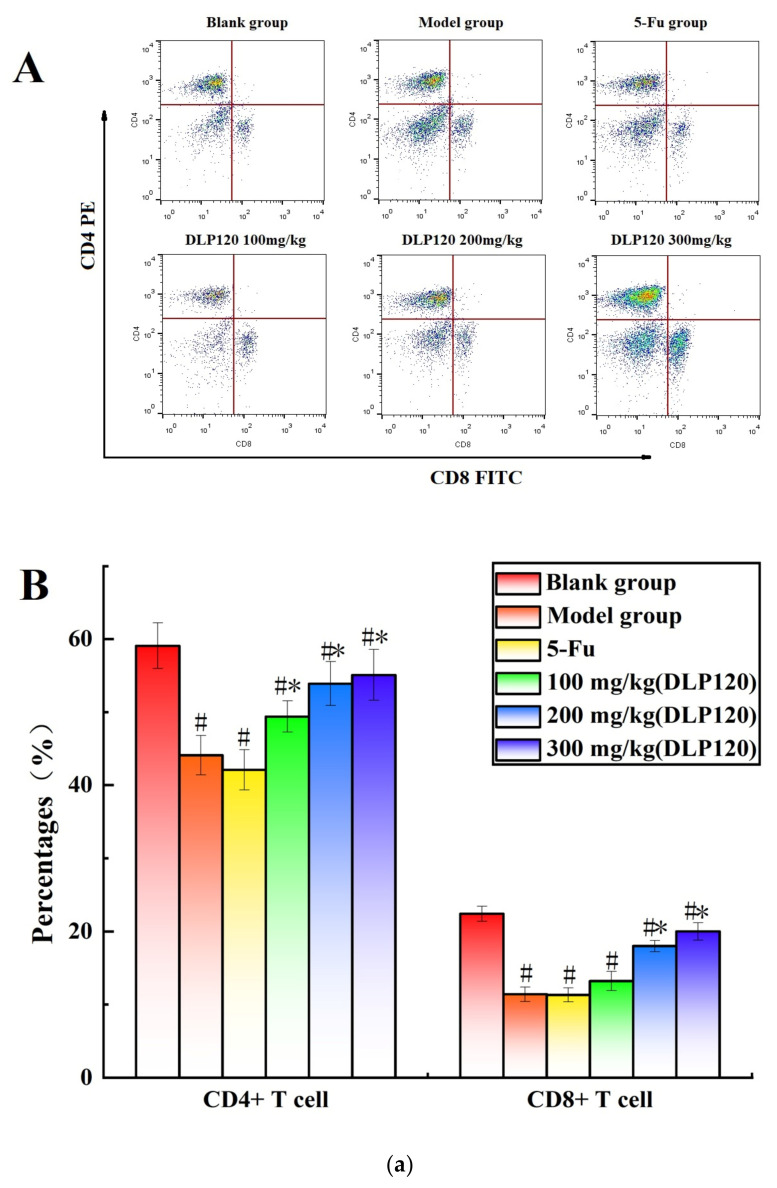

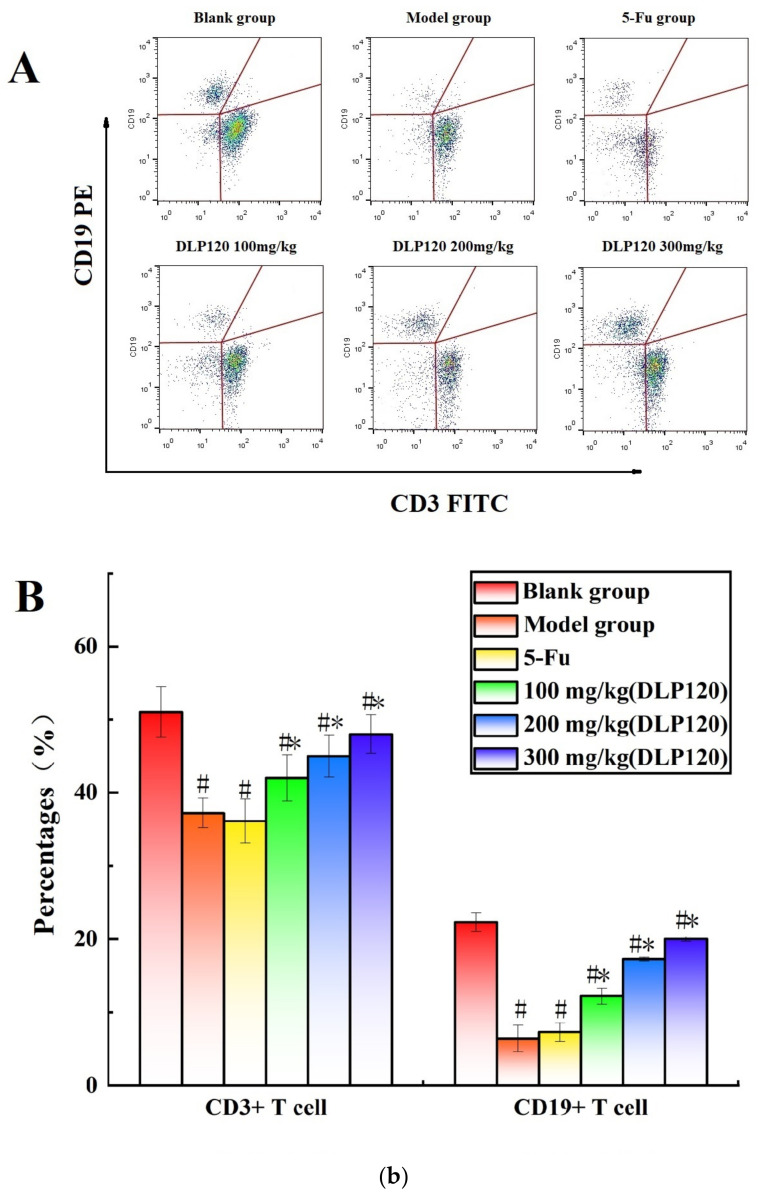
(**a**) The proportion of CD4+ and CD8+ in peripheral blood at the different treatment groups (A) and a bar graph summarizing proportional data on proportion (B). (**b**) Proportion of CD3+ and CD19+ in peripheral blood at the different treatment groups (A), and a bar graph summarizing the proportional data (B). Note: ^#^ *p* < 0.05 compared to blank group; * *p* < 0.05, compared to model group.

To further investigate whether DLP120 enhanced the activity of lymphocytes, the distribution of peripheral lymphocyte subsets was examined. Lymphocytes are a vital cell population of the immune system, and the number and function of each subpopulation are important for the maintenance of the immune system. The distribution of different subsets in peripheral blood changes with the onset of cancer [25]. CD19+ is a B cell-specific surface molecule that can stimulate B cell proliferation and activation [26]. The molecules CD3+, CD4+ and CD8+ are T lymphocyte surface antigens, which are mainly responsible for cellular immunity, resist viruses and regulate the immune system [27]. Changes in peripheral blood lymphocyte subsets were shown in Figure 4. The proportion of T lymphocytes (CD3+, CD4+, and CD8+) and B lymphocytes (CD19+) of the tumor-bearing mice was markedly lower than that of the blank group (*p* < 0.05), indicating that the tumor disrupted the immune balance of the mice. Compared with the model group, the proportion of CD8+ T cells was not significantly different at a dose of 100 mg/kg, while higher doses of DLP120 (200 and 300 mg/kg) dramatically increased the proportion of CD4+, CD8+, CD3 and CD19+ subsets, and especially CD19+ T cells. As in the model group, the lymphocyte subsets of the 5-Fu group did not change observably. Together, these results suggest that intragastric administration of DLP120 can increase the proportion of lymphocytes in a dose-dependent manner, thereby enhancing the immunity of tumor-bearing mice and inhibiting tumor growth.

### 3.5. Effect of DLP120 on Solid Tumor and Tumor Cells

**Figure 5:**
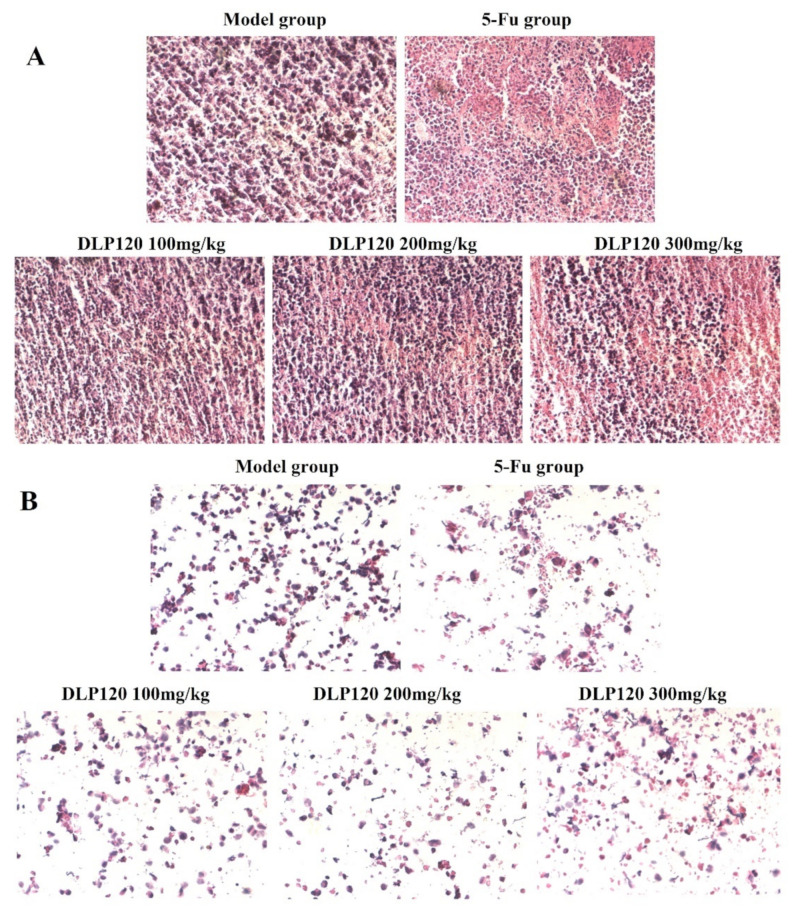
HE staining of slices of H22 tumor tissue (**A**) and tumor cells (**B**).

HE staining was used to observe the condition of tumor tissue and cells. HE stains the nucleus blue-violet, while the cytoplasm and extracellular matrix are stained different shades of red. As shown in Figure 5A, the solid tumor cells in the model group had intact nuclei and normal cell morphology stained purple by hematoxylin, indicating that tumor cells could proliferate normally. Conversely, typical pathological features appeared in the tumor tissues of the 5-Fu group and DLP120 group. The area dyed pink expanded as the dose of DLP120 increased, in which some cells were destroyed, exposing the cytoplasm and producing nuclear debris. As for the 5-Fu group, a large area of tumor tissue was stained pink because of chromatin condensation and shrinking nuclei. In addition, as shown in Figure 5B, cells from ground tissue of the model group were mostly stained purple, with little cell debris. Compared with the model group, the morphology of the DLP120 group was visibly destroyed, and there was distinct nuclear fragmentation. A similar condition was observed in the 5-Fu group due to severe immunosuppressive side effects. The results further verify that DLP120 has a positive effect on inducing the death or apoptosis of tumor tissue cells.

### 3.6. Effect of DLP120 on Tumor Cell Cycle Distribution

**Figure 6:**
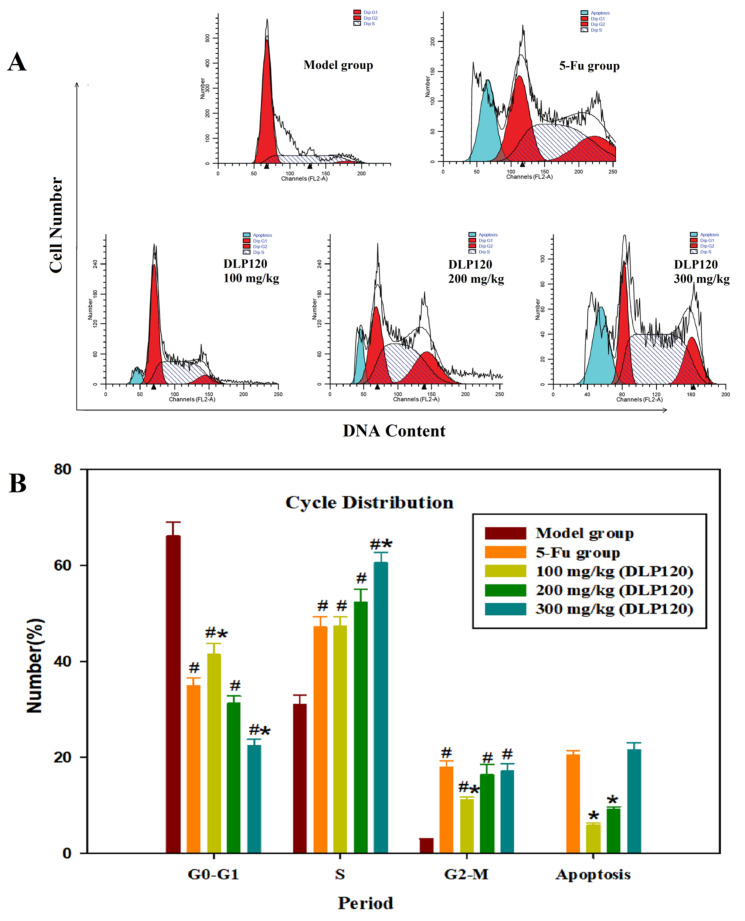
Tumor cell cycle proportions (**A**). Bar graph summarizing the data on cell cycle distribution (**B**). Note: ^#^ *p* < 0.05,compared to model group; * *p* < 0.05, compared to 5-Fu group.

The above experiments demonstrated that DLP120 can protect immune tissues and enhance the activity of the immune system. To further explore the effect of DLP120 on tumor growth inhibition, the cycle distribution of tumor cells was examined. It could be seen from Figure 6 that the cells in the DLP120 group were mainly distributed in the S phase. Compared with the model group, the G1 phase was dramatically decreased in the DLP120 group and the 5-Fu group, while the S phase and the G2 phase were significantly increased, respectively. In addition, the apoptosis rate in the 5-Fu group (20.56%) was not markedly different from that in the DLP120 group at the high-dose (20.46%). This shows that the tumor cell cycle was blocked in the S phase and G2 phase, and the S phase was dominant. It follows that DLP120 may induce apoptosis of tumor tissue by arresting the cell cycle.

### 3.7. Effect of DLP120 on Cell Apoptosis Detection

**Figure 7:**
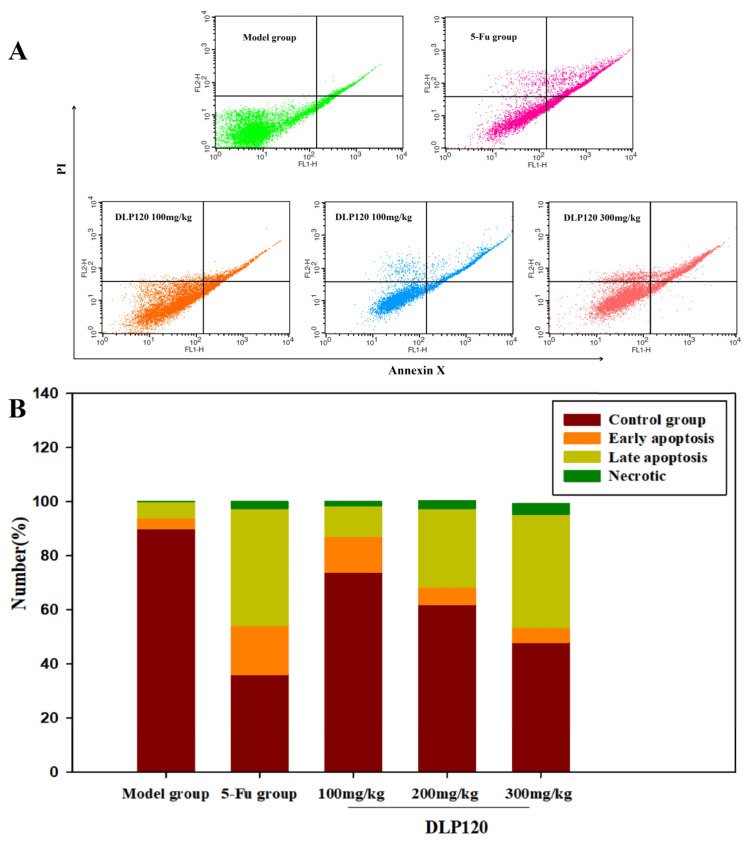
Distribution (**A**) and proportions (**B**) of tumor cell apoptosis.

To accurately confirm that cell cycle arrest was related to apoptosis, cell apoptosis was detected by Annexin V-FITC and PI double staining. As shown from fluorescence distribution (Figure 7), the cells of the model group were mainly normal tumor cells, with only a small amount of early and late apoptotic cells. After administration with different doses of DLP120, early apoptosis decreased by 13.31, 6.39, and 5.48%, respectively. In contrast, late apoptosis increased by 11.27, 29.03, and 41.9% in a dose-dependent manner, respectively. Compared with the DLP120 group at dose of 300 mg/kg, the apoptosis rate of 5-Fu was slightly higher, and late apoptosis dominated. These data show that DLP120 can induce tumor cell apoptosis. The inhibitory effect of DLP120 on tumor growth *in vivo* may be related to tumor cell apoptosis.

### 3.8. Effect of DLP120 on Reactive Oxygen Species

**Figure 8:**
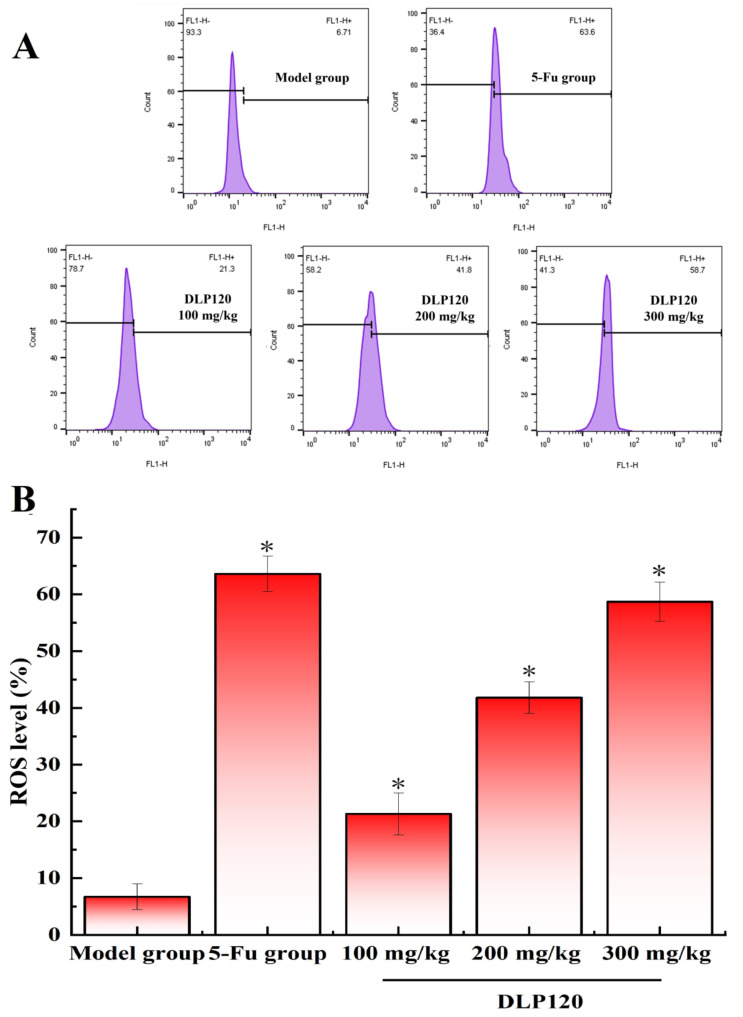
ROS levels (**A**) and a representative bar graph (**B**) of ROS levels in tumor cells. Note: * *p* < 0.05, compared to 5-Fu group.

Many studies have confirmed that reactive oxygen species are associated with the development and progression of certain tumors [28,29]. An excessive increase in ROS causes loss of redox balance, which induces apoptosis and necrosis. To explore if the apoptosis of tumor cells is related to the changes of ROS in the tumor cells, reactive oxygen species in tumor tissue cells were measured. As shown in Figure 8, the DLP120 group significantly increased the production of intracellular ROS in a dose-dependent manner by 21.3, 41.8, and 58.7%. A similar phenomenon occurred in the 5-Fu group. It follows that DLP120 can markedly promote the excessive accumulation of ROS in tumor tissue cells causing oxidative stress, which is extremely likely to stimulate signaling pathways induce apoptosis.

### 3.9. Western Blot Analysis

**Figure 9:**
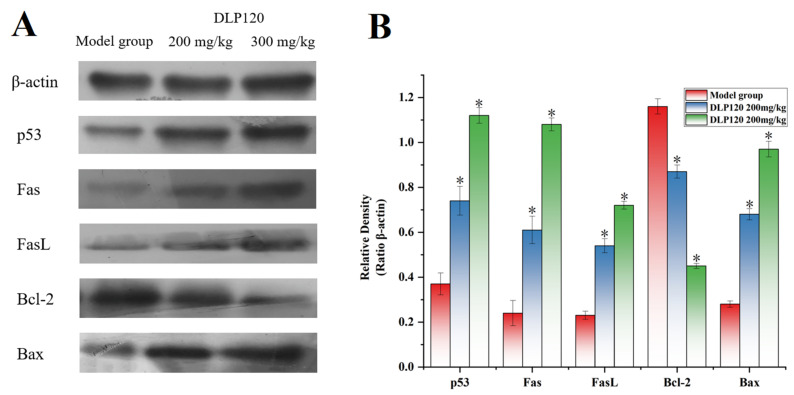
Expression of p53, Bax, Bcl-2, Fas, FasL and β-action of the model group and DLP120 group (**A**), and quantitative analysis (**B**). Note: * *p* < 0.05, compared to model group.

Protein expression related to the p53 pathway was detected by Western blot analysis to evaluate the apoptosis pathway of tumor cells. Figure 9 showed that the DLP120 group dramatically up-regulated p53 protein compared to the model group. Moreover, the expressions of the pro-apoptotic protein Bax was up-regulated, while the anti-apoptotic protein Bcl-2 was down-regulated with the administration of DLP120. In addition, the DLP120 group activated the p53-dependent Fas/FasL apoptotic pathway due to higher expression levels of Fas and FasL. These data further suggest that DLP120-induced tumor cell apoptosis occurs through a ROS-mediated p53 apoptotic pathway.

## 4. Discussion

In a previous study, we obtained a novel dandelion leaf polysaccharide (DLP120), composed of pectin and arabinogalactan, at 120 °C. Pectin has rarely appeared in dandelion polysaccharides reported so far, though pectin and arabinogalactan are considered to have good anticancer potential [30,31]. We found that DLP120 can effectively inhibit the proliferation of HepG2 cells and induce their apoptosis *in vitro*. In this study, we established an H22 tumor-bearing mice model to explore the mechanism of action of DLP120 *in vivo*. We verified that DLP120 can significantly inhibit tumor growth. In addition, DLP120 can also effectively protect immune organs from damage from tumor transplantation. DLP120 alleviated liver and spleen enlargement and attenuated thymus atrophy in H22-transplanted tumor-bearing mice. Immune organs are an important part of the immune system, not only promoting the production and maturation of immune cells, but also carrying immune responses to antigens [32]. Immune cells play a crucial role in controlling tumor growth, which can specifically recognize and eliminate tumors without harming normal cells [33,34]. T lymphocytes and NK cells are lymphoid immune cells [35]. T and B cells induce adaptive immunity, and can specifically recognize almost any foreign antigen. In contrast, NK cells, as innate lymphocytes, are non-antigen-recognition agents and can respond rapidly. It had been reported that the proliferation of NK cells is positively correlated with good prognosis in various solid tumors [36]. Our data showed that DLP120 administration not only markedly enhanced NK cell viability, but also stimulated lymphocyte proliferation and increased lymphocyte subsets ratios. Thus, DLP120 can protect and enhance lymphocyte function and attenuate immune system dysfunction brought by tumors, thereby inhibiting tumor growth *in vivo.*

To further explore the mechanism of tumor growth inhibition, HE staining was used to observe tumor tissues and cells, and morphological characteristics of apoptosis appeared. It was found that ROS significantly increased with DLP120 in a dose-dependent manner. Excessive accumulation of ROS can disrupt the redox balance of the microenvironment and cause cell damage [33]. Severe cell damage can activate p53. Furthermore, the p53 signaling pathway is one of the important pathways regulating various cellular functions such as DNA repair, apoptosis, cell senescence and the cell cycle [37]. The results of WB experiments also revealed that p53 protein was activated and further activated the expression of the pro-apoptotic protein Bax, and inhibited the expression of the anti-apoptotic protein Bcl-2. Notably, we found that Fas and FasL proteins associated with death receptors were up-regulated. High expression of p53 can activate Fas [38]. Fas and Fas L (Fas ligand) are interacting pro-apoptotic receptor/ligand pairs that can trigger the death receptor apoptotic pathway [39]. It has been reported that Fas-induced apoptosis plays an important role in controlling tumor progression [40]. Overall, DLP120 can trigger the p53 signaling pathway to induce tumor cell apoptosis to inhibit growth *in vivo*.

## 5. Conclusions

**Figure 10:**
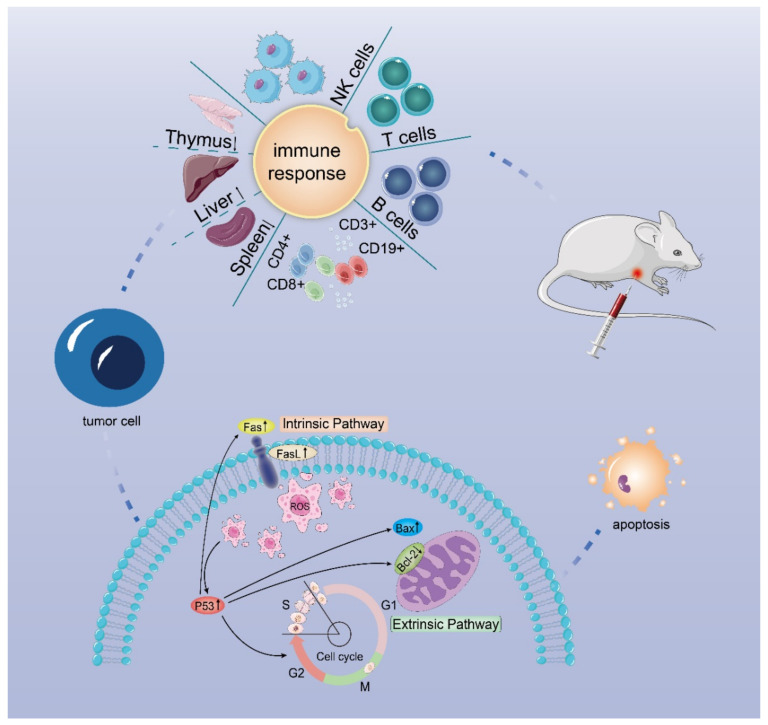
Possible mechanism of action of DLP120 in H22 tumor-bearing mice.

In conclusion, a pectin-arabinogalactan polysaccharide (DLP120) isolated from dandelion leaf can effectively enhance the immune function of tumor-bearing mice and induce tumor cell apoptosis to inhibit tumor growth. Our data show that administration of DLP120 resulted in a significant accumulation of ROS in tumor cells, thereby arresting cell cycle in S and G2 phases, and activating the p53 signaling pathway. Bax was up-regulated while Blc-2 was down-regulated. In addition, p53 also activated Fas and the ligand FasL, triggering the death receptor apoptotic pathway. Figure 10 shows the antitumor mechanism of DLP120 in H22 tumor-bearing mice. Our results, on the one hand, provide an experimental basis for a dandelion leaf polysaccharide in the treatment of hepatocellular carcinoma in the future and, on the other hand, provide theoretical support for this polysaccharide as a new type of functional health food and drug adjuvant. However, there are still some unresolved problems in this study. For example, the types of lymphocyte proliferation induced by Con A and LPS. The antitumor activity of the dandelion polysaccharide was only obtained in a limited animal model, and we need to find new mechanisms ,targets and structure-activity relationship to ensure its efficacy. 

## Figures and Tables

**Table 1:** Effects of DLP120 on organs indexes of H22 tumor-bearing mice.

Groups	Mice Body Weight (g)		Numbers Start/End	Thymus Index (mg/g)	Spleen Index (mg/g)	Liver Index (mg/g)	Tumor Weight(g)/ Tumor Inhibition Rate(%)
	Start	End					
Blank	20.32 ± 1.02	30.98 ± 1.58	10/10	3.56 ± 0.31	3.30 ± 0.54	53.72 ± 4.65	-
Model	19.87 ± 0.97	32.32 ± 1.49	10/10	1.64 ± 0.56 ^#^	8.58 ± 1.06 ^#^	67.06 ± 5.31 ^#^	3.74/-
5-Fu (20 mg/kg)	19.56 ± 1.24	24.19 ± 0.87 ^#,^*	10/8	2.14 ± 0.37 ^#,^*	6.79 ± 0.97 ^#,^*	79.29 ± 6.35 ^#,^*	1.20/67.95
DLP120 (100 mg/kg)	20.31 ± 1.05	32.16 ± 1.89	10/10	2.02 ± 0.29 ^#,^*	8.12 ± 1.24 ^#^	69.87. ± 6.18 ^#^	3.04/18.75
DLP120 (200 mg/kg)	20.63 ± 0.97	31.44 ± 1.64	10/10	3.11 ± 0.41 ^#,^*	6.42 ± 0.89 ^#,^*	68.20 ± 5.63 ^#^	1.89/49.52
DLP120 (300 mg/kg)	19.74 ± 0.87	30.12 ± 1.63	10/10	3.83 ± 0.59 *	5.57 ± 1.12 ^#,^*	67.22 ± 4.51 ^#^	1.51/59.64

Note: ^#^ *p* < 0.05 compared to blank group; * *p* < 0.05 compared to model group; ”-“ stands for that it did not exist.

## Data Availability

The date are available from the corresponding author.
